# Complete inhibition of phosphatase and tensin homolog promotes the normal and oxygen-glucose deprivation/reperfusion-injured PC12 cells to cell death

**DOI:** 10.15171/jcvtr.2018.13

**Published:** 2018-05-21

**Authors:** Sohrab Minaei Beyrami, Mohammad Hasan Khadem Ansari, Yousef Rasemi, Nader Shakib, Pouran Karimi

**Affiliations:** ^1^Department of Biochemistry, Faculty of Medicine, Urmia University of Medical Sciences, Urmia, Iran; ^2^Neurosciences Research Center, Tabriz University of Medical Sciences, Tabriz, Iran

**Keywords:** OGD, Reperfusion Injury, AKT, p38, MAPK, PC12 Cells

## Abstract

***Introduction:*** Lipid phosphatase and tensin homolog deleted from chromosome 10 (PTEN) antagonizes phosphoinositide 3-kinase (PI3K)/AKT cell survival pathway. The effect of PTEN inhibitors has been rarely examined on cell survival following reperfusion injury. In this study, we investigated the neuroprotective effect of SF1670, as a new PTEN inhibitor, on an in vitro stroke-like model.

***Methods:*** PC12 cells were exposed to oxygen-glucose deprivation/reperfusion (OGD/R). The cells were treated in five conditions as follows: normoxic normoglycemic (NO/NG); 60 minutes OGD; 60 minutes OGD and 6 h reperfusion (OGD/R); OGD/R treated with 10 µM SF1670 (OGD/R-SF), and NO/NG treated with 10 µM SF1670 (NO/NG-SF). Then, phosphorylation levels of AKT, P38 in PC12 cells were measured by immunoblotting. The cell viability was also determined by colorimetric assay.

***Results:*** The results of immunoblotting revealed that following OGD/R the levels of phospho-AKT (p-AKT) significantly decreased, compared to NO/NG cells (*P * < 0.05). However, the ratio of p-AKT/total AKT significantly increased in the presence of SF1670 in the OGD/R-SF group, compared to the OGD/R condition. On the other hand, SF1670 significantly reduced the p-P38 MAPK and p-JNK levels, compared to OGD/R cells. Moreover, cell viability significantly decreased in the OGD and OGD/R condition compared to NO/NG cells. Surprisingly, SF-treated cells (OGD/R-SF and NO/NG-SF group) showed low cell viability compared to NO/NG condition.

***Conclusion:*** Overall, our results demonstrated that complete inhibition of phosphatase activity of PTEN not only did not exhibit neuroprotective effect but also promoted PC12-deprived cells to death.

## Introduction

Ischemic stroke, as a common life-threatening cerebrovascular disease,^1^ causes a high percentage of permanent disabilities all around the world.^2^ The fibrinolytic treatment of ischemic stroke or successful recanalization^3^ restores the oxygenation but initiates secondary local inflammation after reperfusion^3,4^ which in turn exacerbates cerebral tissue injury, the so-called reperfusion injury (I/R).^4,5^ I/R injury initiate widespread inflammation, reactive oxidation, excitotoxicity and cell-specific dysregulation of metabolic processes promoting neurodegeneration through specific programmed cell death mechanisms.^6^

studies have proven that the balance between two intracellular signaling pathways including the phosphatidylinositol 3-kinase/AKT (PI3K/AKT) as a cell survival pathway, and mitogen-activated protein kinase (MAPK) as an inflammatory pathway^7^ play important role in the determination of cell fate after I/R.^8^ Different mechanisms which are involved in the inhibition or enhancement of these pathways could be taken as suitable therapeutic targets to limit I/R damages.^9^ MAPKs family is consist of p38 group of protein kinases, c-jun N-terminal (c-JNK), and extracellular signal-regulated kinases (ERKs). P38 MAPK pathway, as a major stress kinase, activates through phosphorylation in responses to cellular stress, heat shock, oxidative stress, and inflammation.^10,11^ A research has revealed that activation of P38 initiates apoptosis cascade and up-regulates pro-inflammatory cytokines production.^12^ In addition, C-JNK activation is also associated with neuronal death.^13-15^

In contrary, phosphorylated AKT (p-AKT) in downstream phosphorylates numerous substrates are involved in cell growth,^16^ proliferation,^17^ and survival,^18^ as well as cellular metabolism, glucose uptake and angiogenesis and protects cells from I/R induced cell injury.^19^

Phosphatase and tensin homolog (PTEN) is a lipid phosphatase and tumor suppressor which plays an important role in the regulation of cell proliferation, differentiation, and apoptosis.^20,21^ Up-regulation of PTEN is capable to inhibit AKT activation by degradation of PI3P to PIP2^16,21^ and facilitates the p38 MAPK signaling pathway.^22,23^ Decreasing PTEN activity through phosphorylation of PTEN^24^ or deleting of one copy of its gene increases resistance to apoptotic cell death.^25^ Hence, down-regulation of PTEN may lead to a neuroprotective effect in the neurodegenerative disorders with a beneficiary role in I/R injury.^26-28^

The current paper used a recently developed specific PTEN inhibitor, SF1670,^29^ for inhibiting PTEN pathway.^30,31^ Li et al showed that pretreatment with SF1670 in nanomolar concentration enhances PIP3 signaling in transplanted neutrophils.^29^ In the current study, for the first time, we examined the effects of pretreatment with high dose (10 µM) of SF1670 for complete inhibition of phosphatase activity of PTEN against I/R injury in oxygen-glucose deprivation (OGD) as an in vitro stroke-like model in PC12 cultured cells.

## Materials and Methods

### Chemicals and antibodies

PC12 cells were obtained from Pasteur Institute (Tehran, Iran), DMEM (Gibco, Grand Island, NY,USA), p-Akt1/2/3 Antibody (Ser 473): sc-7985 (SANTA CRUZ, CA, USA), p-p38 Antibody (Tyr 182): sc-101759 (SANTA CRUZ, CA, USA), p38α Antibody (N-20): sc-728 (SANTA CRUZ, CA, USA), p-JNK Antibody (14.Thr 183/Tyr 185): sc-293136 (SANTA CRUZ, CA, USA), JNK1/3 Antibody (C-17): sc-474, RIPA Buffer (Sigma-Aldrich, New York, NY, USA), Anti-Protease Cocktail (Sigma-Aldrich, New York, NY, USA), Acrylamide and bisacrylamide (Sigma-Aldrich, New York, NY, USA).

### Cell culture

Rat pheochromocytoma-derived cell line PC12 cells were cultured in normoxic normoglycemic (NO/NG) condition as following: The cells were seeded in six-well plate in Dulbecco’s modified Eagle’s medium (DMEM) (Gibco, Grand Island, NY, USA) supplemented with 10% horse serum, 5% fetal bovine serum (FBS), 100 kU/L of penicillin, and 100 mg/L of streptomycin (Sigma, St. Louis, MS, USA) and maintained at 37^◦^C in a normoglycemic (5 mM glucose) and humidified normoxic atmosphere incubator (95% air and 5% CO2). All treatments were performed on cells at 80% confluence.

### Oxygen-glucose deprivation/reperfusion model

In order to mimic ischemic-like conditions in vitro, PC12 cells were exposed to OGD/R injury as following: the culture media of the cell, DMEM, was replaced by Hanks Balanced Salt (HBSS; glucose concentration = 0 mg/dl) and then transferred to a hypoxic chamber (95% nitrogen and CO2 5%) for 60 minutes. At the end of the OGD phase, the cells reperfused for 6 h in normoxic-normoglycemic (NO/NG) condition.^32,33^ The pretreatment was performed by 10 µM of SF1670 24 hours before OGD induction (OGD/R-SF group). We also pre-treated a batch of normoxic and normoglycemic cells by SF1670 as drug control (NO/NG-SF group).

### Western blotting

PC12 cells were subjected to Western blot analysis for phosphorylation of p38, and AKT proteins as previously described^34^ with minor changes. Briefly, 10^6^ cells were homogenized in 500 µl lysis buffer [0.05 mmol/L Tris-NaOH (pH = 8), 150 mmol/L NaCl, 0.01 mmol/L EGTA, 1%SDS, 0.1% anti Protease Cocktails (ROCHE)]. The supernatants were mixed with loading buffer solution containing 60 mM Tris-HCl, 25% glycerol, 2% SDS, 14.4 mmol/L 2-mercaptoethanol, and 0.1% bromophenol blue. Then proteins were separated on a 10% SDS-polyacrylamide gel and transferred onto the nitrocellulose membrane. After incubation in blocking buffer (phosphate buffered saline, 3% (w/v) BSA, 0.1% Tween 20), the membranes were probed overnight at 4°C with the appropriate primary antibody as follows: rabbit polyclonal anti-phospho-Akt, anti-AKT, anti-phospho-p38, anti-p38, anti-phospho-JNK, anti-JNK antibodies. Having washed and exposed to horseradish peroxidase-conjugated secondary antibody for 1 hour at room temperature, antibody-antigen complexes were visualized by enhanced chemiluminescence substrates. The scanned images of the protein bands were analyzed using ImageJ (National Institutes of Health, Bethesda, Maryland, USA) software.

### Cell viability assay

Cell viability was determined using the 3-[4, 5-dimethylthiazol-2-yl]-2, 5-diphenyltetrazolium bromide (MTT). PC12 cells (2-5 × 10^5^ cells/well) were seeded in 96-well plates in DMEM medium until confluence 80%. Following the interventions, MTT reagent (20 µl, 5 mg/ml in PBS) was added to wells and incubated for 4 h at 37°C. Then the medium was removed, and replaced with 150 µl DMSO. Afterward, absorbance was measured at 570 nm by an automatic microplate reader (Awareness Technologies Stat Fax 4200).

### Statistical analysis

Data was analyzed using SPSS software version 16.0 (SPSS, Chicago, IL, USA) and expressed in mean ±SD. One-way ANOVA and Tukey’s post-hoc tests were used in statistical comparisons. *P* values less than 0.05 were considered as significant.

## Results

### The effect of SF1670 on AKT phosphorylation in OGD/R-injured PC12 cells

**Figure 1:**
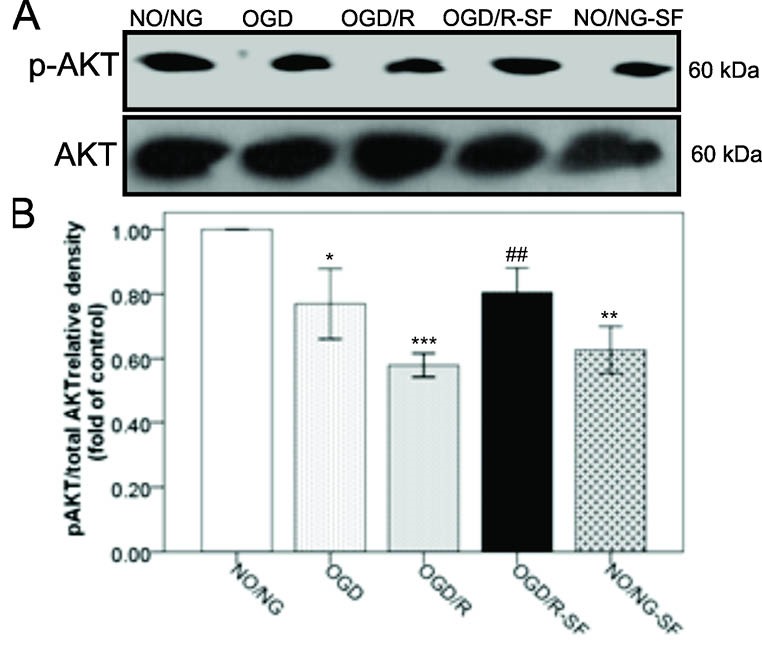
The effect of SF1670 on AKT phosphorylation in PC12 cell exposed to OGD/R. **A)** Immunoblotting images of p-AKT and total AKT proteins in PC12 cells exposed to one hour OGD followed by 6 h of reperfusion and/or 10µM SF1670 pretreatment. **B**) Quantitative densitometric analysis of the p-AKT against total AKT protein bands. Data are presented as means ± SEM. *P <0.05, **P < 0.01, ***P < 0.001 vs. NO/NG. ##*P* < 0.01 vs. OGD/R. [NO/NG: normoxic/normoglycemic cells; OGD: Oxygen and glucose deprived cells (60 min); R: Reperfused cells for 6 hours; SF: SF1670 (10 µM) treated cells with SF1670 one hour before OGD].

Figure 1 shows that AKT phosphorylation levels (p-AKT) significantly (*P *< 0.05) decreased in the OGD-treated cells, compared to NO/NG cells. Moreover, reperfusion (6 hours) following OGD significantly (*P *< 0.001) decreased p-AKT/total AKT ratio in the OGD/R cells, compared to NO/NG cells. Conversely, SF1670 pretreatment (24 hours before OGD/R) significantly (*P *< 0.01) increased p-AKT/total AKT ratio, compared to the OGD/R condition. Surprisingly, SF1670 (*P *< 0.01) decreased the phosphorylation of AKT in normoxic normoglycemic cells (NO/NG-SF), compared to the NO/NG cells.

### The effect of SF1670 on p38 MAPK phosphorylation in OGD/R-injured PC12 cells

**Figure 2:**
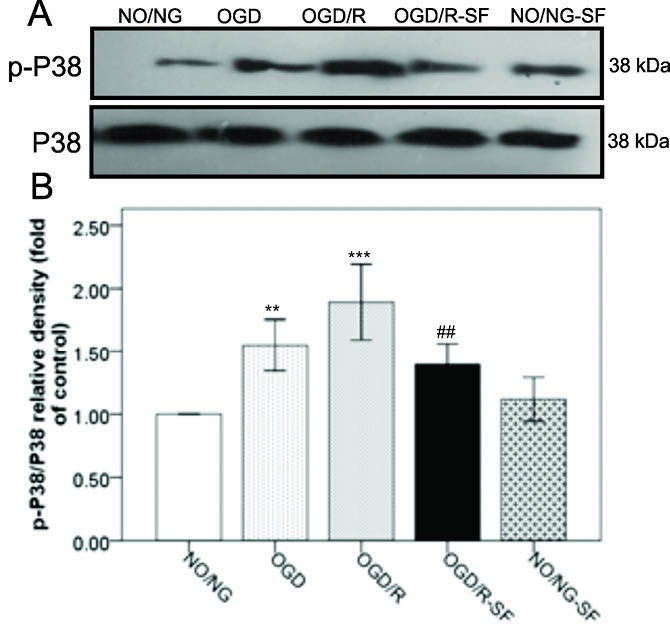
The effect of SF1670 on phosphorylation of P38 MAPK in PC12 cell exposed to OGD/R. **A)** Immunoblotting images of p-P38 MAPK and total P38 MAPK proteins in PC12 cells exposed to one hour OGD followed by 6 h of reperfusion and/or 10µM SF1670 pretreatment. **B**) Quantitative densitometric analysis of the p- P38 MAPK against total P38 MAPK protein bands. Data are presented as means ± SEM. **P<0.01, ***P<0.001 vs. NO/NG. ##*P*<0.01 vs. OGD/R. [NO/NG: normoxic/normoglycemic cells; OGD: Oxygen and glucose deprived cells (60 min); R: Reperfused cells for 6 hours; SF: SF1670 (10 µM) treated cells with SF1670 one hour before OGD].

Figure 2 shows a significant increase in the average levels of phospho-P38 MAPK (p-P38 MAPK) in the OGD (*P *< 0.01) and OGD/R (*P *< 0.001) conditions, compared to the NO/NG condition. Nevertheless, SF1670 administration in OGD/R-SF group significantly (*P *< 0.01) decreased the P38 MAPK phosphorylation, compared to OGD/R cells. No significant change was observed in the levels of p-P38 MAPK in the NO/NG-SF condition, compared to the NO/NG condition.

### The effect of SF1670 on c-JNK phosphorylation after being exposed to OGD /R

**Figure 3:**
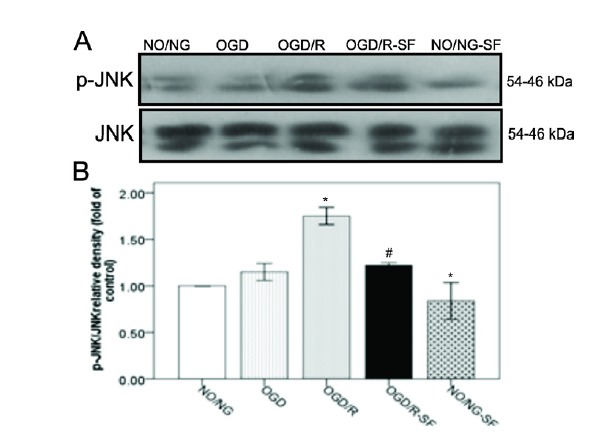
The effect of SF1670 on phosphorylation of JNK in PC12 cell exposed to OGD/R. A) Immunoblotting images of p-JNK and total JNK (as loading control) proteins in PC12 cells exposed to one hour OGD followed by 6 h of reperfusion and/ or 10μM SF1670 pretreatment. B) Quantitative densitometric analysis of the p-JNK against total JNK protein bands. Data are presented as means ± SEM. *P <0.05, vs. NO/NG; #P<0.05 vs. OGD/R. [NO/NG: normoxic/normoglycemic cells; OGD: Oxygen and glucose deprived cells (60 min); R: Reperfused cells for 6 hours; SF: SF1670 (10 μM) treated cells with SF1670 one hour before OGD].

Our results also demonstrated that OGD/R condition significantly (Figure 3, *P *< 0.05) increased phospho-JNK (p-JNK) levels, compared to the NO/NG. However, SF1670 pretreatment in the OGD/R-SF group decreased the p-JNK when compared to OGD/R cells (*P *< 0.05). No significant change was observed in the levels of p-JNK between NO/NG-SF and NO/NG condition.

### The effect of SF1670 on cell viability

**Figure 4:**
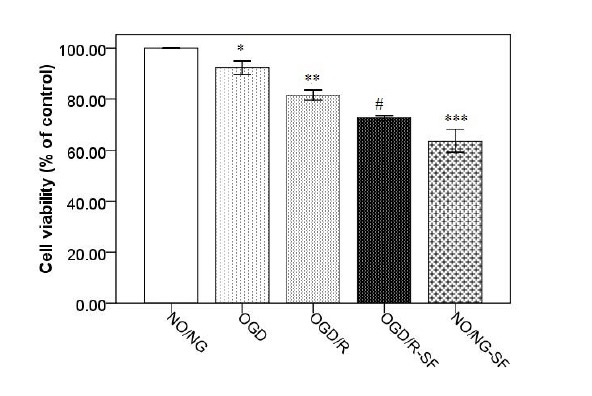
The effect of SF1670 on cell viability (MTT) in cultured PC12 cells exposed to OGD/R insult or OGDR-SF1670. Data are presented as means ± SEM. *P<0.05, **P<0.01, ***P<0.001. vs. NO/NG; #P<0.05 vs. OGD/R. [NO/NG: normoxic/normoglycemic cells; OGD: Oxygen and glucose deprived cells (60 min); R: Reperfused cells for 6 hours; SF: SF1670 (10 μM) treated cells with SF1670 one hour before OGD].

In addition, MTT assay was carried out to evaluate cellular viability in the PC12 cells. The results showed that cell viability was reduced after exposure to OGD for 1 hour (*P *< 0.05), and OGD-R for 6 hours (*P *< 0.01), compared to NO/NG condition (Figure 4). The SF1670 at the concentration of 10 µmol/L decreased (*P *< 0.05) cell viability in OGD/R-SF, compared to the OGD/R. Furthermore, SF1670 decreased the cell viability in NO/NG-SF, compared to NO/NG (*P *< 0.001).

## Discussion

The current paper deprived PC12 cells of oxygen and glucose for 1h followed by 6 hours reperfusion to induce a valid in vitro stroke-like model. The current study aimed at investigating the neuroprotective effect of PTEN inhibitor, SF1670, on OGD/R-induced injury by assessing the phosphorylation of AKT, JNK, and P38 MAPK proteins, and cell viability. The results of the current study showed that SF1670 increased p-AKT, and decreased p-P38, p-JNK, and cell viability in the PC12 cells exposed to OGD/R insult.

The oxygen and glucose deprivation are two common reasons for ischemic insult damages which are deteriorated after reoxygenation.^1,8,10^ PTEN is an upstream negative regulator of AKT signaling pathway which inhibits AKT phosphorylation by PI3K, the positive regulator of AKT. Therefore, PTEN activity is associated with down-regulation of PI3K/Akt down-streams involved in cell survival pathways.^35,36^ Conversion of Inositol diphosphate (IP2) to Inositol 3, 4, 5-triphosphate (IP3) is required to Akt phosphorylation which promotes cellular survival and attenuates cell death. The current paper showed that exposure to OGD and reperfusion reduced p-AKT levels in the PC12 cells. As shown in Figures 1 and 4, the parallel changes were detected for p-AKT expression and cell viability during the experiment. Similarly, in the previous report the OGD/R-induced cell viability loss in PC12 cells was associated with diminished Akt phosphorylation.^37^ In support of our finding, previous studies also showed that ischemia and reperfusion inhibited PI3K/Akt pathway resulting in cell death.^38,39^

P38 and JNK, stress-activated kinases, play important role in the regulation of apoptosis signals. Previous studies demonstrated that JNK and P38 activities were up-regulated in response to the brain ischemia. In addition, these proteins are involved in cerebral ischemia/reperfusion insult, and inhibition of their phosphorylation attenuates ischemic brain injury.^40-42^ Recently, a study also reported that OGD/R increases p-P38 and p-JNK in PC12 cells.^42^ Similarly, in the present experiment, the levels of p-JNK and p-P38 expression were significantly amplified following OGD/R. Nevertheless, SF1670 pretreatment prevented these changes induced by OGD/R. Moreover, our study showed that increased p-P38 MAPK and p-JNK levels were accompanied with cell viability loss in OGD/R. In addition, it has been shown that AKT signaling via phosphorylation of apoptosis signal-regulated kinase 1 (ASK1), inhibits the JNK and p38-mediated apoptosis.^43,44^

In this study, SF1670 treatment attenuated p-AKT, p-p38, and p-JNK accompanied by cell viability loss. Although previous evidence indicated that activation of p38 and JNK is associated with apoptotic cell death, some reports revealed that p38 MAPK plays a critical role in the control of cell survival and proliferation.^45-47^ Phong et al have demonstrated that p38 signaling promotes cell survival in response to DNA damage possibly to inhibit the onset of premature apoptosis.^48^ Moreover, previous reports linked p38 signaling pathway to increased levels of antiapoptotic protein such as Bcl-2 and Bcl-x_l_ following DNA damage and stress.^49,50^ It seems that the role of p38 signaling in the control of apoptosis is context dependent and depending on the physiological context of the stress induction it may switch from cell survival to pro-apoptosis. In the present study, SF1670 attenuated p-p38 and p-JNK in PC12 cells accompanied by low cell viability. We suggest that ameliorated JNK and P38 MAPK signaling pathways promote cells toward cell death.

Furthermore, we found that pretreatment of NO/NG cells with SF1670 attenuated p-AKT expression and cell viability as assessed by MTT. We suggest that SF1670 in 10 µM concentration has a neurotoxic effect on PC12 cells. In addition, it is likely that other apoptosis-prompting factors override the cytoprotective effects of SF1670 activity. This paradoxical effect of 10 µM of SF1670 may be related to the dual roles of PTEN in the cell. Lately, Zhou et al. reported that inhibition of PTEN with bpV(HOpic) aggravates ischemic acute kidney injury via augmenting apoptosis and inflammation.^51^ It has also reported that astrocytic PTEN loss exacerbated ischemia damage.^52^ Evidence has also shown that PTEN-knockout mice died in early development.^16^ Therefore, it is likely that the complete suppression of phosphatase activity of PTEN could not be a good idea in cell protection, particularly in this model which was made by neuroblastoma cells. Probably PTEN has other crucial roles in cell viability that have not been clarified yet which needs further studies. Similar to other novel protein, the specific substrates of PTEN are a mystery. To identify of this phosphatase, systematic approaches including generation of null mutations, exploration of possible roles in transient overexpression studies, further studies are needed to be given mutants with normal PTEN activities.

In summary, the study demonstrated that complete inhibition of phosphatase activity of PTEN promoted cells toward death, possibly through attenuation p38 signaling pathways in OGD/R PC12 cells.

## Ethical approval

The ethical approval for this study was obtained from ethics committee of Tabriz University of Medical Sciences.

## Competing interests

None.
